# Supplementary material for: Towards unified AI-driven fracture mechanics: the extended deep energy method (XDEM)
Source: Nat Commun. 2026 Aug 15;17:8492. doi: 10.1038/s41467-026-76748-1 (PMC13484471; doi:10.1038/s41467-026-76748-1)
Supplement: Supplementary file 3 [file 41467_2026_76748_MOESM3_ESM.zip › pic/Results/Stress_intensity_factor/Mode1/crack1.pdf]

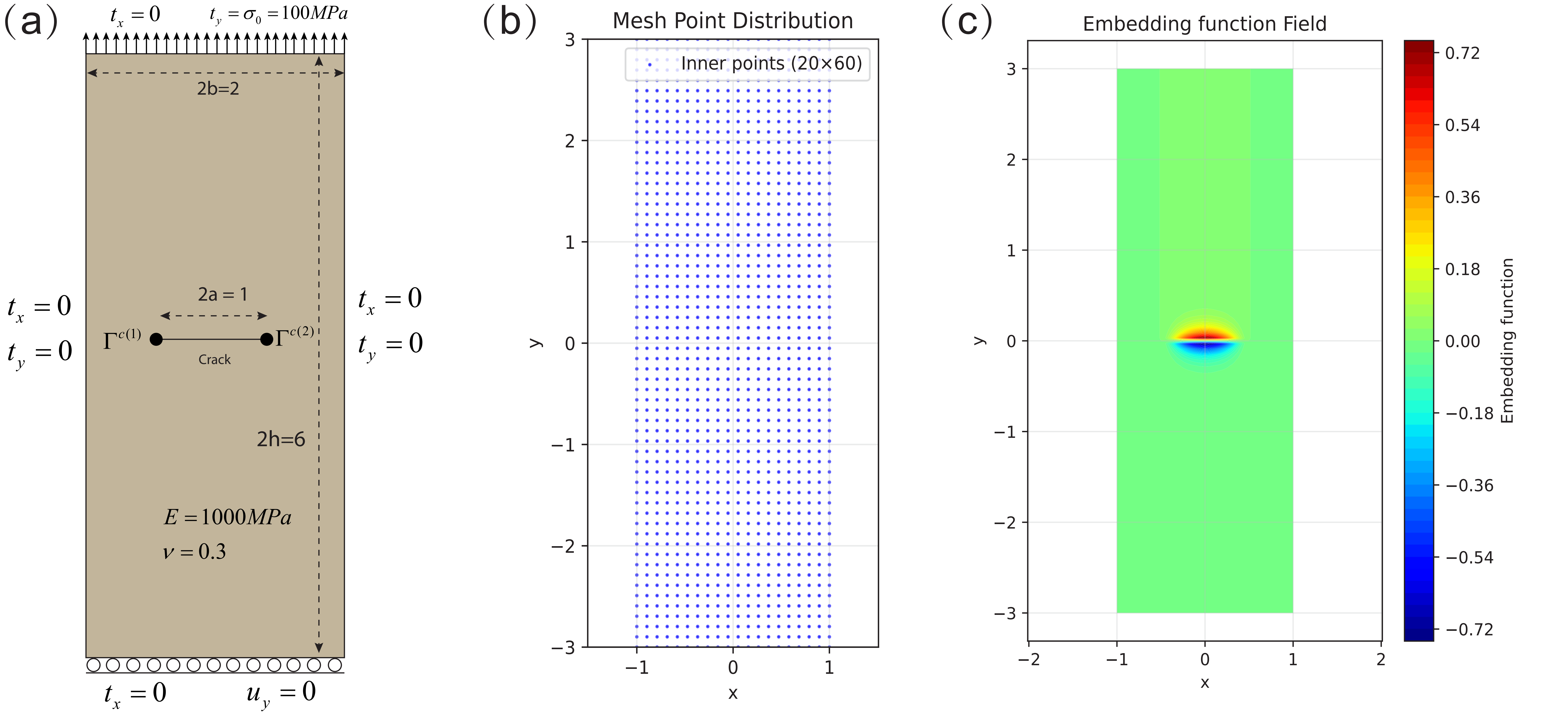
(a)
$t_x = 0$
$t_y = \sigma_0 = 100MPa$
2b=2
2a = 1
$t_x = 0$
$t_y = 0$
$\Gamma^{c(1)}$
$\Gamma^{c(2)}$
Crack
$t_x = 0$
$t_y = 0$
2h=6
$E = 1000MPa$
$\nu = 0.3$
$t_x = 0$
$u_y = 0$
(b)
Mesh Point Distribution
Inner points (20×60)
y
x
(c)
Embedding function Field
Embedding function
y
x
